# Supplementary material for: Impact of multi-echo ICA modeling decisions on motor-task fMRI analysis
Source: Apert Neuro. Author manuscript; Available in PMC 2026 Oct 15. (PMC13662799; doi:10.52294/001c.162891)
Supplement: Supplementary file 1 [file NIHMS2215239-supplement-Reddy_et_al_2026_-_Supplemental_Figures.pdf]

Supplemental Materials

Supplemental Table 1. Scan parameters for functional scans acquired in each dataset.

Dataset	Volumes	TR (s)	TE (ms)	FA (°)	MB factor	GRAPPA	Voxel size (mm^3)
**Healthy Hand**	210	2	10.8/28.03/45.26/62.49/79.72	70	4	2	2.5x2.5x2
**Healthy Shoulder**	250	2.2	13.4/39.5/65.6	90	2	2	1.731x1.731x4
**MSHand**	258	1.5	10.6/27.83/45.06/62.29/79.52	70	4	2	2.5x2.5x2.5
**MSFoot**	324	1.5	10.6/27.83/45.06/62.29/79.52	70	4	2	2.5x2.5x2.5

A

Dataset	# scans with task-correlated components / # total scans	% variance explained by task-correlated components
HealthyHand	7 / 16	2.8 ± 1.1
MSHand	2 / 14	1.1 ± 0.1
MSFoot	4 / 14	1.3 ± 0.3
HealthyShoulder	8 / 28	5.6 ± 6.0

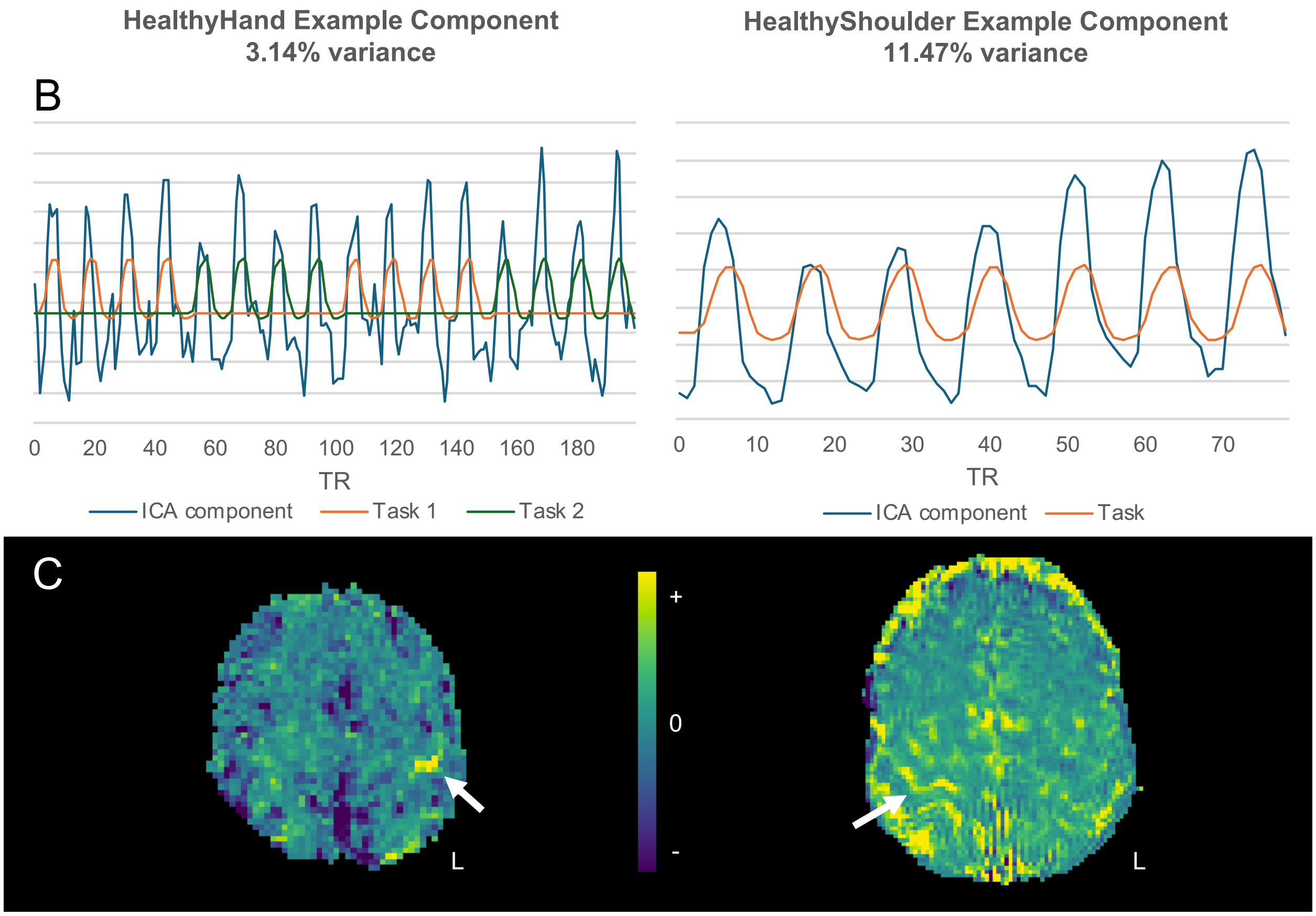

Supplemental Figure 1. Task-correlated components identified during ME-ICA classification were ‘accepted’ in the Moderate model. (A) Summary of scans with identified task-correlated components across datasets; in each of these scans, 1 or 2 components were identified. Two examples of task-correlated components are displayed, including (B) time course compared to task regressor(s) and (C) spatial maps. White arrows indicate regions of expected motor activity.

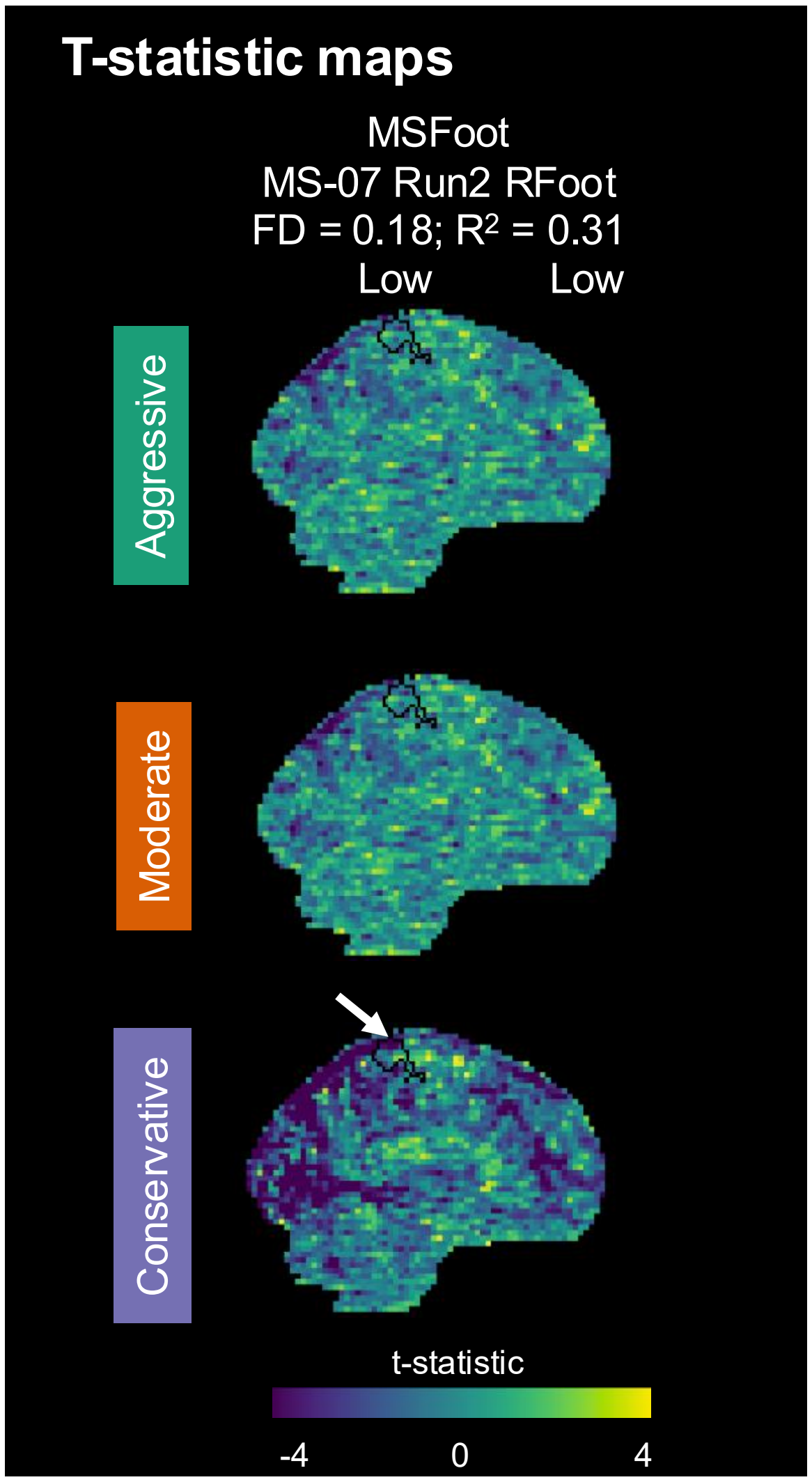

Supplemental Figure 2. Example MSFoot scan demonstrates artifactual negative t-statistics that are retained with the Conservative model. The primary motor cortex ROI is shown in black outline.

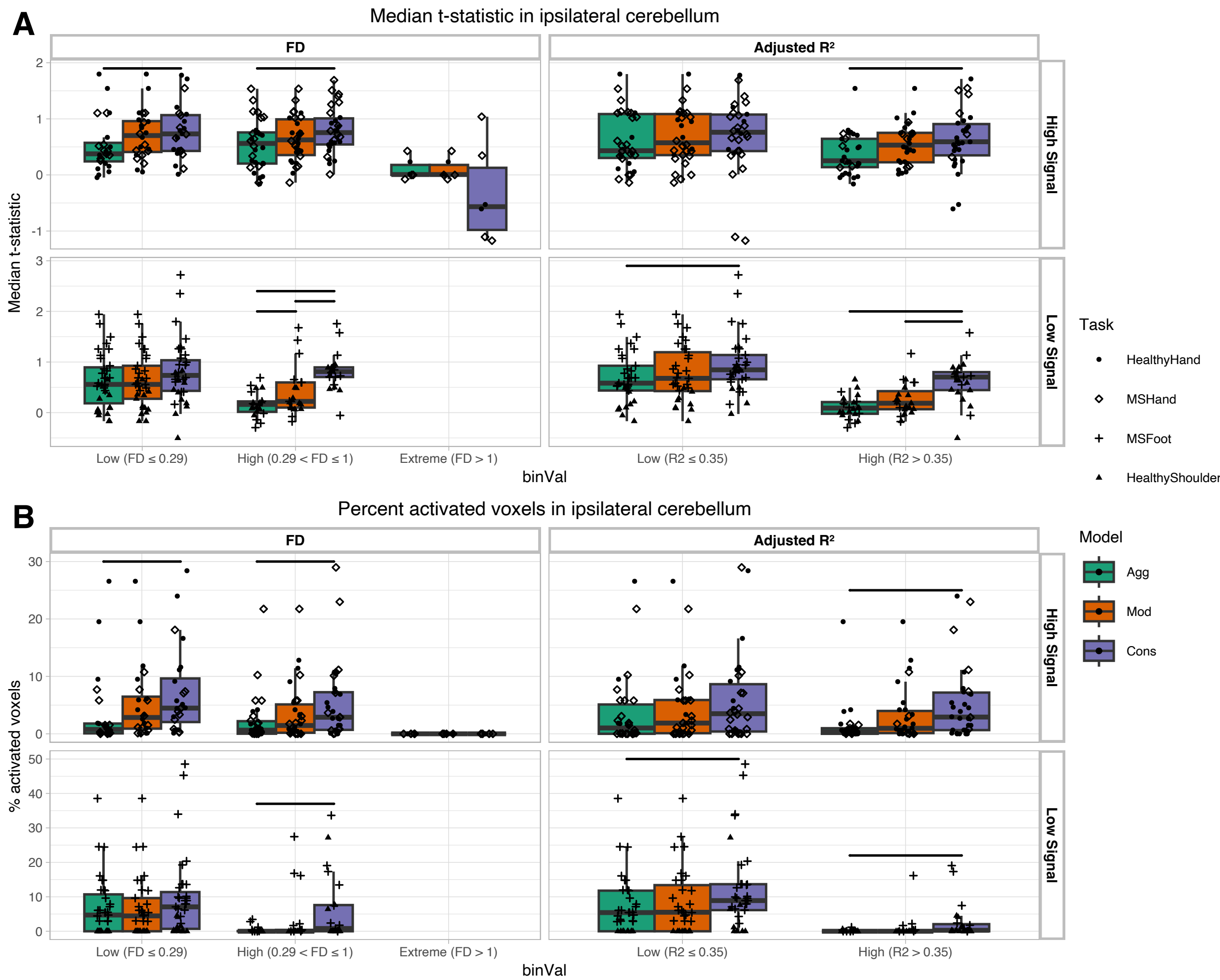

Supplemental Figure 3. Activation metrics calculated in significant group-level regions within the ipsilateral cerebellum. Regions were determined as the union of significant regions across all three models. (A) Median t-statistic and (B) Percent activated voxels were calculated for each scan. Horizontal bars indicate significant differences between models ($p < 0.05$, Bonferroni-corrected).

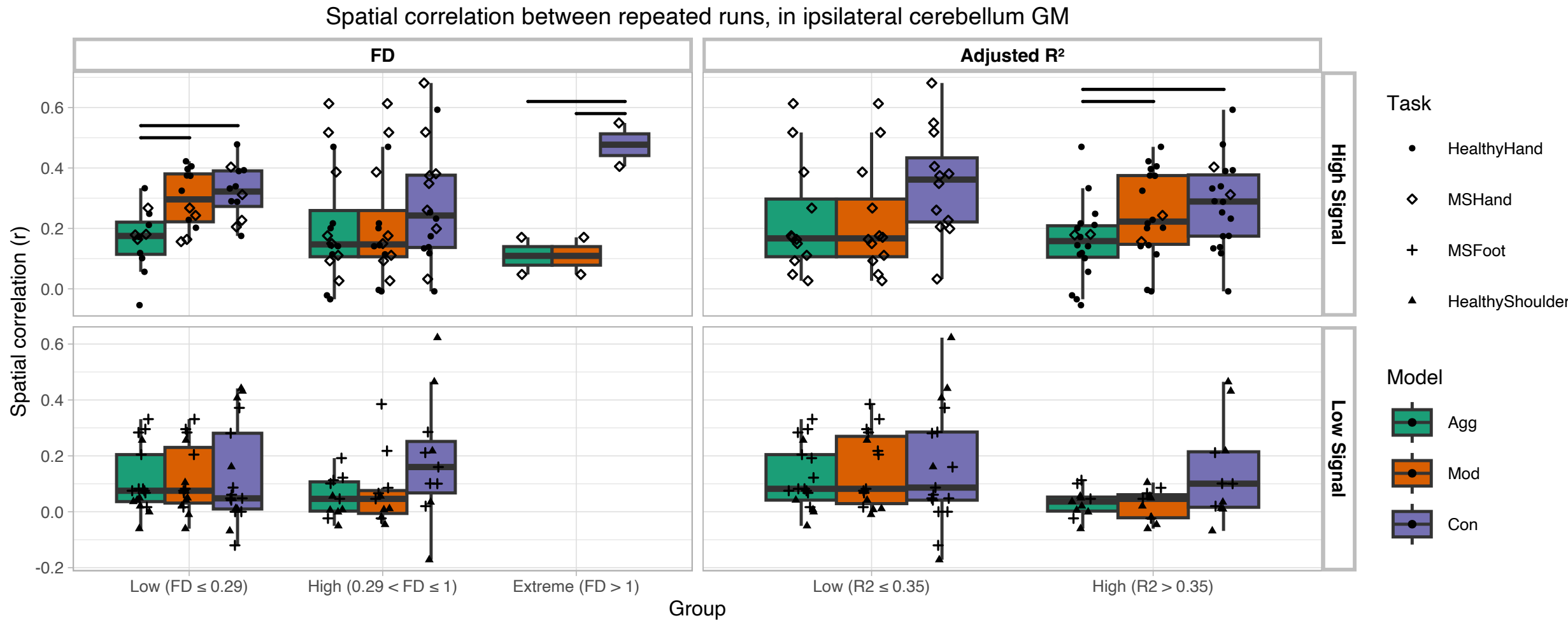

Supplemental Figure 4. Spatial correlation between runs 1 and 2 for each dataset and model, calculated in gray matter (GM) of the ipsilateral cerebellum. Horizontal bars indicate significant differences between models ($p < 0.05$, Bonferroni-corrected).
